# Tissue engineering using 3D printed nano-bioactive glass loaded with NELL1 gene for repairing alveolar bone defects

**DOI:** 10.1093/rb/rby015

**Published:** 2018-07-31

**Authors:** Jing Zhang, Yang Chen, Jing Xu, Jingjing Wang, Chengzhang Li, Liyan Wang

**Affiliations:** 1 Department of Periodontology, School and Hospital of Stomatology, Wuhan University, Wuhan, Hubei, China; 2 Department of Stomatology, Foshan Woman and Children’s Hospital, Foshan, Guangdong, China; 3 Department of Spinal Surgery, Shenzhen Second People’s Hospital, Shenzhen, Guangdong, China; 4 Department of Stomatology, Jiangyin People’s Hospital of Southeast University, Jiangyin, Jiangsu, China

**Keywords:** bone tissue engineering, nano-bioactive glass, Nel-like type I molecular-1, bone marrow mesenchymal stem cells, bone regeneration

## Abstract

The purposes of this study were to construct a novel tissue engineered bone composed of 3D-printed bioactive glass block/chitosan nanoparticles (BD/CSn) composites loaded with Nel-like Type I molecular-1 DNA (pDNA-NELL1) and/or bone marrow mesenchymal stem cells (BMSCs), and study their osteogenic activities by repairing bone defects in rhesus monkeys. CSn with NELL1 gene plasmid and rhesus monkey BMSCs were composited with a BD scaffold to prepare the tissue-engineered bone. Four adult female rhesus monkeys with 10- to 12-years old and 5–7 kg in weight were used in animal experiments. The first and second premolar teeth from four regions of each monkey were removed to form bone defects with size of 10 × 10 × 5 mm, which were then implanted with above-mentioned tissue engineered bone. At 12 weeks after the implantation, gross observations, X-ray and micro-CT observations revealed that the new bone was extremely close to normal bone in mass, density, hardness, and structure. The bony cortex was smooth and closely connected to the surrounding normal bone. Histological observations revealed moderate inflammation in the repair area, and the new bone tissues were similar to normal ones. In conclusion, tissue engineered bone of this study exhibited good osteoconductivity for promoting the formation of new alveolar bone tissue, and NELL1 gene played a promotional role in bone regeneration.

## Introduction

One problem in stomatology is the repair the bone defects caused by various reasons (e.g. trauma, tumor and periodontal disease). Autologous bone transplantation is the gold standard for the repair of large area of bone defects [1, 2]; however, surgical grafting causes a new wound on the donor site, which leads to secondary trauma and pain to the patient. Rejection and secondary infection in allograft transplantation induces bone graft failure [3, 4]. Artificial bone grafting materials (e.g. metal alloys and polymers) are not fully satisfactory regarding biocompatibility, infection risks, rejection responses and biological or mechanical properties. The theory of tissue engineering by combining medical principles with engineering technology provides better therapeutic solutions for the repair of large bone defects. This is achieved through inoculating seed cells obtained from *in vitro* culture and amplification to an absorbable and degradable scaffold with good biocompatibility and inducing the proliferation and differentiation of cells with growth factors [5–7].

Nel-like Type I molecular-1 (NELL1) is a secretory protein composed of 810 amino acids, and is an osteoinductive protein that controls skeletal ossification. Overexpression or lack of NELL1 could induce several bone diseases [8]. Previous studies found that bone regeneration was promoted in polyethylene particle-induced osteolysis in NELL1 transgenic mice compared with normal mice [9]. When NELL1 was delivered to whole body of osteoporotic mice, bone mineral density would increase; local delivery of NELL1 to spine of osteoporotic sheep led to significant increase in bone formation [8]. The applications of NELL1 in bone tissue engineering are being explored. Animal experiment has demonstrated NELL1 contained biomaterial could promote the repair of cranial bone defect. Moreover, the biological effects of NELL1 only available for the cells with osteogenic potential, rather than fibroblasts [10]. Death rate and teratogenic rate of NELL1 transgenic mice were both lower than those of bone morphogenetic Protein 2 transgenic mice [11]. The quantity and quality of NELL1 induced bone tissue were similar to the natural bone [12].

This study tends to provide a more effective grafting material for the clinical repair of large bone defect area by constructing the artificial bone graft material with a composite scaffold of 3D-printed bioactive glass (BG) and chitosan nanoparticles (CSn). In order to construct a tissue engineered bone, CSn was loaded with NELL-1 DNA (pDNA-NELL1), and bone marrow mesenchymal stem cells (BMSCs) were seeded on the scaffold as seed cells. The tissue-engineered bone was implanted into the bone defect area of the alveolar socket of rhesus monkeys to evaluate the *in situ* osteogenesis ability. Gross observation, x-ray and micro-CT observations were employed for observing the repair effects on large bone defects in the alveolar sockets of the rhesus monkeys.

## Materials and methods

### Preparation of alveolar tissue-engineered bone

#### Scaffold preparations

The 3D-printed BG used in this study was provided by the National Engineering Research Center for Tissue Restoration and Reconstruction (NERC-TRR), South China University of Technology.

**Figure 1:**
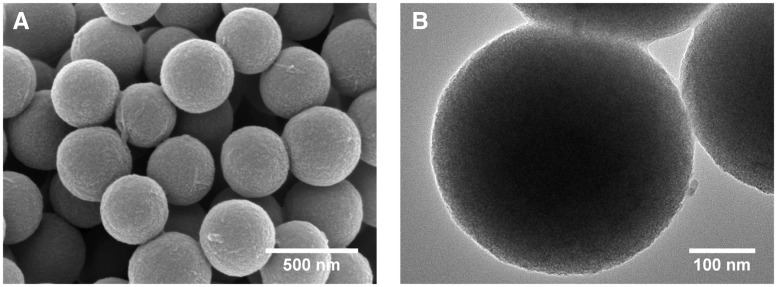
SEM micrograph **(A)** and TEM micrograph **(B)** of the 3D-printed porous bioglass scaffold

3D printed BG is a type of porous scaffold prepared by fiber deposition technology with BG microspheres (350 nm in diameter) as printing materials and PVA as a binder. The scaffolds were prepared according to previously published literatures [13, 14]. Briefly, 3D printing was performed by a 3D-Bioprinter (Hangzhou Regenovo Biotechnology Co., Ltd., China) at room temperature to construct a cuboid BG scaffold of 10 × 10 × 5 mm with pore size of 250 μm. The 3D-printed porous scaffold was then immersed into high-concentration PBS to form K_3_CaH(PO_4_)_2_, which enhances the compressive strength of the scaffold. The microstructure of the scaffold was observed by scanning electronic microscopy (SEM) and transmission electron microscopy (TEM), as shown by Fig. 1. Homogeneous BG microspheres were combined together to form porous structure with connected pores. Such porous structure was contribute to transportation of nutrition and metabolic waste, thus promoting tissue regeneration.

#### Extracting and culturing BMSCs

Six-year-old healthy adult female rhesus monkeys weighing 3–5 kg were selected for iliac bone marrow puncture. A 8–10 ml marrow was extracted and transferred to a flask, and then cultured in DMEM/F12 medium with 10% fetal bovine serum and 1% penicillin-streptomycin double antibiotic in a cell incubator at 37°C with 5% CO_2_. The BMSCs in the marrow grew adhering to the wall of the culture flask. The separation of BMSCs from the marrow was observed via a microscope each day. The cell culture medium was replaced every 2 days, and the cells were passaged every 3 days. The BMSCs were rinsed with PBS before replacing the medium or cell passage, to remove detached cells and metabolic wastes. After 1 week, the culture medium was replaced every 3 days. When the BMSCs covered 80% of the bottom of the culture flask, 0.05% trypsin was used for digestion and subsequent steps. The third and fourth generations of the cells were collected for preparing the tissue-engineered bone.

#### Preparation of growth factor pDNA-NELL1 nanoparticles carried by chitosan

A 10 g chitosan was dissolved in 360 ml 1-methyl-2-pyrrolidone and added with 35 ml NaOH (15%, aq) and 57.5 ml CH_3_I while stirring. After stirring for 2 h, the solution was poured into ethanol for sedimentation. The sediment was dissolved with NaCl and placed in a dialysis bag. Dialysis was carried out within 1 M NaCl for 4 days and then washed by tri-distilled water for 3 days (changed twice daily). Starch test paper was used to detect iodine residue, and the sediment was dried by lyophilization, labeled as TMC (trimenthyl chitosan). 1 mg TMC was dissolved in 1 ml bone salivary protein (BSP) to prepare TMC solution with a concentration of 1 mg/ml. Then 0.4 ml of pDNA-NELL1 was dissolved in 1.2 ml above-mentioned TMC-BSP solution to prepare DNA solution with a concentration of 0.5 mg/ml. The mixed solution was shaken vigorously using a Vortex (2500 rpm) for 30 s immediately after mixing, and then placed in a 37°C water bath for 30 min, then the nanoparticle solution loaded with pDNA-NELL1 with an N/*P* values of 10 was prepared.

#### Construction of tissue-engineered graft materials

Four types of graft material were constructed for animal experiments: BD (3D printed BG), BD+BMSCs, BD\CSn + BMSCs, and BD\CSn(pDNA-NELL1) + BMSCs. For each type, BD scaffold was pretreated by sterilization using UV radiation and washed by PBS solution for five times, 3 min at a time; then the BD scaffold was immersed into low glucose DMEM within a 50-ml tube for pre-wetting overnight.

Before the implantation, 1 mg/ml chitosan solution and BMSCs suspension with 5 × 10^6^ cells/ml were prepared, then the graft materials were prepared as follows.

- BD: 220 μl saline was added to 3D printed BG scaffold and infiltrated into the scaffold for 4–6 h, then 200 μl saline was added to the scaffold.
- BD+BMSCs: 220 μl saline was added to 3D printed BG scaffold and infiltrated into the scaffold for 4–6 h, then 200 μl BMSCs suspension was added to the scaffold.
- BD\CSn + BMSCs: 200 μl chitosan solution and 20 μl saline were mixed with vibration for 30 s, then the mixture was added to the 3D printed BG scaffold and infiltrated into the scaffold for 4–6 h, followed by adding 200 μl BMSCs suspension to the scaffold.
- BD\CSn(pDNA-NELL1) + BMSCs: 200 μl chitosan solution and 20 μl above mentioned pDNA-NELL1 solution were mixed with vibration for 30 s, then the mixture was added to the 3D printed BG scaffold and infiltrated into the scaffold for 4–6 h, followed by adding 200 μl BMSCs suspension to the scaffold.

For above graft materials 2–4, 1 × 10^6^ cells were seeded on each scaffold. The implantation was performed immediately after the preparation of each scaffold.

### Animal experiment

#### Experimental animals and grouping

The animal experiments were approved by the Ethical Committee of Foshan Woman and Children’s Hospital. Four adult female rhesus monkeys, aged between 10 and 12 years and weighed 5–7 kg were purchased from the Guangdong Primate Research Center. The monkeys exhibited complete dentition, healthy periodontal tissue and teeth, and were free of obvious systemic diseases. The four rhesus monkeys were randomly labeled as A, B, C and D, and their (upper, lower, left and right) jaws were labeled as 1, 2, 3, 4, respectively according to the international oral area. Thus, there was a total of 16 jaws from the group of four monkeys. The first and second premolar teeth of the four regions were removed to form 16 bone defect areas measuring 10 × 10 × 5 mm. The four types of graft materials (BD, BD+BMSCs, BD\CSn + BMSCs and BD\CSn(pDNA-NELL1) + BMSCs) were implanted to ensure that there were four materials in each jaw region of each rhesus, so as to eliminate the differences between the different monkeys and jaw regions. The observation period was 12 weeks.

The animal experiments set three control groups (1, 2 and 3) and one experimental group. Control group 1: 3D printed BG block (BD); control group 2: 3D printed BG block loaded with BMSCs (BD + BMSCs); and control group 3: 3D printed BG block/chitosan composite load with BMSCs (BD\CSn + BMSCs). The experimental group used 3D printed BG block/chitosan composite loaded with BMSCs and NELL1 gene (BD\CSn(pDNA-NELL1) + BMSCs) (Fig. 1).

#### Experimental procedures

##### Establishment of the alveolar bone defect model

**Figure 2:**
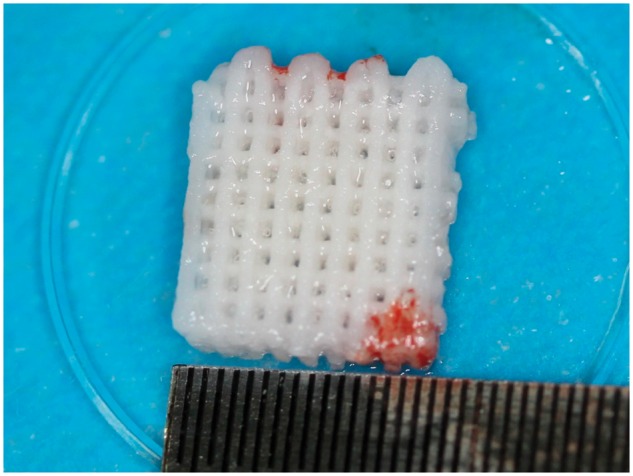
The 3D-printed BG porous scaffold composited with NELL1 loaded CSn and BMSCs

The animals fasted from water and food for 24 h prior to the operation, and were administered general anesthesia with 3% pentobarbital sodium via a slow intravenous bonus injection. Following disinfection, the first and second premolar teeth from each jaw were removed, where the gum was also separated using a gum detacher. Buccal mucoperiosteal flap of the surgical area was turned over; and a dental high-speed hand piece was used to remove the peripheral socket bone and buccal bone cortex, as well as intact lingual bone cortex was retained to prevent the loss of material. A bone defect area of ∼10 × 10 × 5 mm was established for each site, and the cancellous bone was exposed. The defect area was treated with dental high-speed hand piece again to ensure that the surface of the defect area was smooth and clear, and matched the material size. The bone defect area was washed with saline (Fig. 2A and B).

##### Biological material implantation and postoperative care

**Figure 3:**
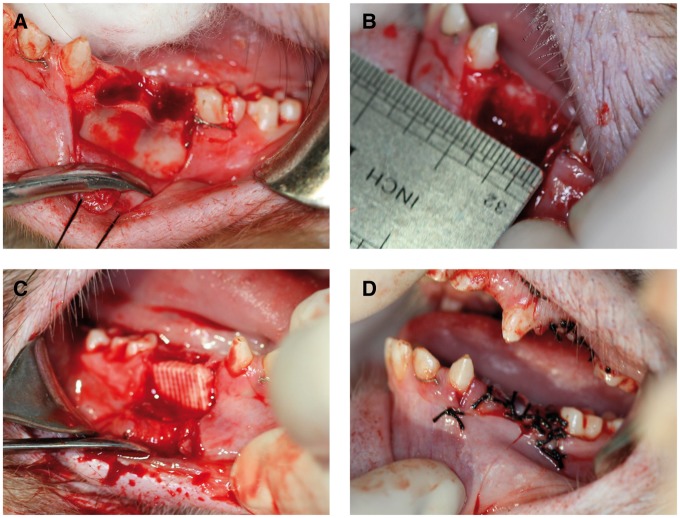
**(A)** The first and the second premolar teeth were removed; **(B)** a cuboid alveolar bone defect model of 10 × 10 × 5 mm was created by removing the buccal bone cortex; **(C)** the bone defect area filled with biological materials; **(D)** the opposite suture of the gingival flap

For both experimental group and control groups, the tissues at the defect areas were rinsed with saline and the bone debris were removed before the implantation. After the implantation (Fig. 2C), the graft materials were completely covered by buccal mucoperiosteum and fixed using gingival restoration and apposition suture (Fig. 2D). Following gingival suture and local disinfection, the animals were fed with soft food for 2 weeks after the operation. To prevent systemic infection, 1 g ceftriaxone sodium and 4 mg dexamethasone were administered via an intramuscular injection for 7 consecutive days, and 50 ml of 2% chlorhexidine was used twice daily for 2 weeks for oral rinsing and surgical site disinfection. The wound was closely observed for signs of inflammations (e.g. redness, swelling or exudation) and complications (e.g. suppuration and necrosis), and the suture was removed 2 weeks later if the abovementioned conditions were absent (Fig. 3).

##### Post-operative observation

The following conditions were primarily observed: Abnormal daily diet, activities, mood, inflammation (e.g. redness, swelling or exudation) and complications (e.g. suppuration and necrosis).

**Figure 4:**
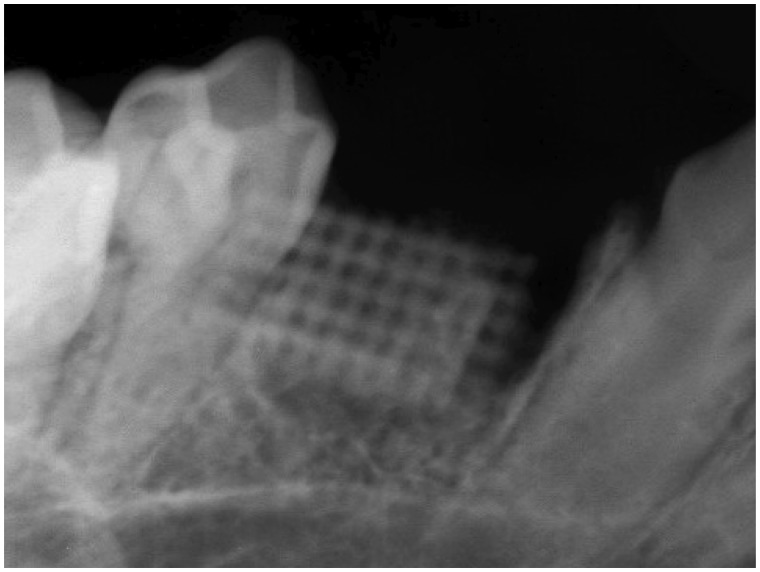
X-Ray examination of the scaffold immediately post-operation

X-ray examinations were performed immediately post-operation to verify correct implantation of the tissue engineered bone into the bone defect area, as shown in Fig. 4.

Gross observation by naked eye, X-ray observations, and micro-CT 3D reconstruction of the grafting area were carried out 12 weeks after the implantation. All the jaw samples containing the repair regions were obtained from the rhesus. Before taking the specimen, the animals were treated similar to the procedures prior to the implantation. For each animal, the canine teeth of each jaw were removed following disinfection. A trapezoid incision was made using a scalpel at the sampling location. A gum detacher was used to separate the buccal and lingual mucoperiosteal flap in the surgical region. A high-speed dental hand piece was used to expand and take the specimen, which was a bone block of 15 × 12 × 7 mm. Each specimen was labeled immediately after the sampling. The specimen was fixed in a 10% formaldehyde solution for 7 days.

## Results

### Gross observations

**Figure 5:**
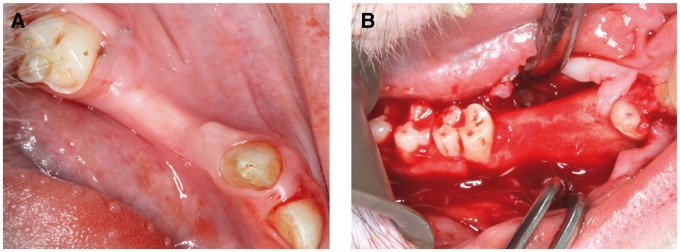
Bone regeneration of the experimental group before **(A)** and after flap **(B)**

New bone formation was observed by naked eye on the specimens. The size of the new bone was similar to that of the implanted materials, and the boundary between the new bone and the surrounding normal bone tissue was not obvious. The new bone exhibited a smooth surface, without obvious depression or protruding, and was covered by periosteum. Bone regeneration could be directly observed in the grafting area (Fig. 5A and B). Alveolar crest was relative plump without obvious depression. After flap, the interface between the regenerated bone and the host bone was blurred, and the surface was covered with periosteum.

### X-ray observations

**Figure 6:**
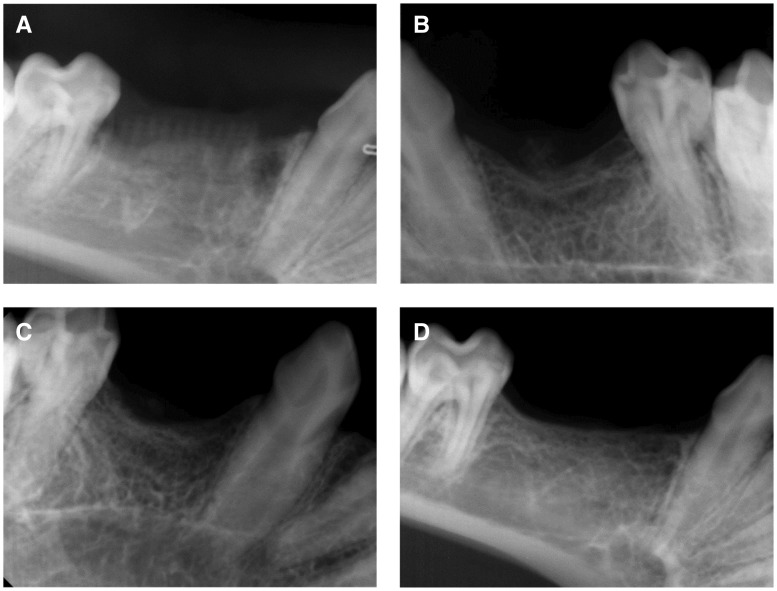
X-ray observations of bone regeneration for the experimental group and the control groups. **(A)** BD group, **(B)** BD + BMSCs group, **(C)** BD\CS + BMSCs group, **(D)** BD\CS (pDNA-NELL1) + BMSCs group)

X-ray observations of each group are shown in Fig. 6. An x-ray of the experimental group (BD\CSn(pDNA-NELL1) + BMSCs) is presented in Fig. 6D, suggesting an unclear boundary between the bone graft materials and the surrounding bone. The bone graft materials were almost completely absorbed and reconstructed. A large amount of regenerated trabecular bone was observed, which was clearly arranged in a porous network structure uniformly. The regenerated bony cortex was homogeneous and continuous. The alveolar crest was higher than those of the control groups; however, it was similar to that of the peripheral host bone. There was a wide range of high-density radiodensity shadow in the grafting region, indicating mature bone tissues formed in the regenerated region.

A representative X-ray observation from control Group 1 (BD) is shown in Fig. 6A, showing a clear boundary between the grafting materials and the surrounding bone. The more similar the materials used for absorption and reconstruction were to the surrounding bone, the better the observed regenerative effect. There scattered loose bone trabecula and grafting materials were clearly observed near the alveolar crest.

A representative X-ray observation from control Group 2 (BD + BMSCs) is shown in Fig. 6B, showing an unclear boundary between the grafting materials and the surrounding bone. The bone graft materials were almost completely absorbed and reconstructed. The trabecula was clear in structure, less in quantity, and exhibited a scattered arrangement. The alveolar crest was U-shaped, and the most concave region was lower than the surrounding host bone.

A representative X-ray observation from control Group 3 (BD\CSn + BMSCs) is shown in Fig. 6C, showing an unclear boundary between the grafting materials and surrounding bones. The bone graft materials were almost completely absorbed and reconstructed. The trabecula was clear in structure, fewer but thicker than the BD + BMSCs group, and exhibited a scattered arrangement. The alveolar crest was U-shaped, and the most concave region was lower than the surrounding host bone. The radiodensity shadow was darker in the grafting region than that of the BD + BMSCs group, indicating better osteogenesis effect of the BD\CSn + BMSCs group than the BD + BMSCs group.

### Micro-CT 3D reconstruction

**Figure 7:**
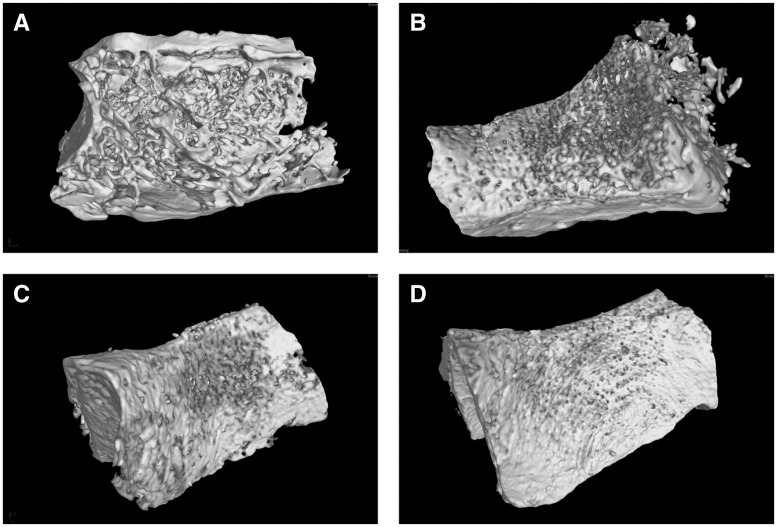
Micro-CT 3D reconstructions of bone regeneration for the experimental group and the control groups. **(A)** BD group, **(B)** BD + BMSCs group, **(C)** BD\CS + BMSCs group, **(D)** BD\CS (pDNA-NELL1) + BMSCs group)

Figure 7 shows micro-CT 3D reconstruction model of each group. The models revealed that new bone formed in both the experimental and the control groups. Compared with the control groups, the trabecula of the experimental group was thicker, denser and more closely arranged (Fig. 7D). In the BD group, the trabecula was disorganized and sparsely formed with cavities (Fig. 7A). The trabecula was clear in structure with large quantities in both BD + BMSC and BD\CSn + BMSC group without cavities. The new bone was integrated with the surrounding bone without obvious boundary, and the bone defect healed well (Fig. 7B and C).

**Figure 8:**
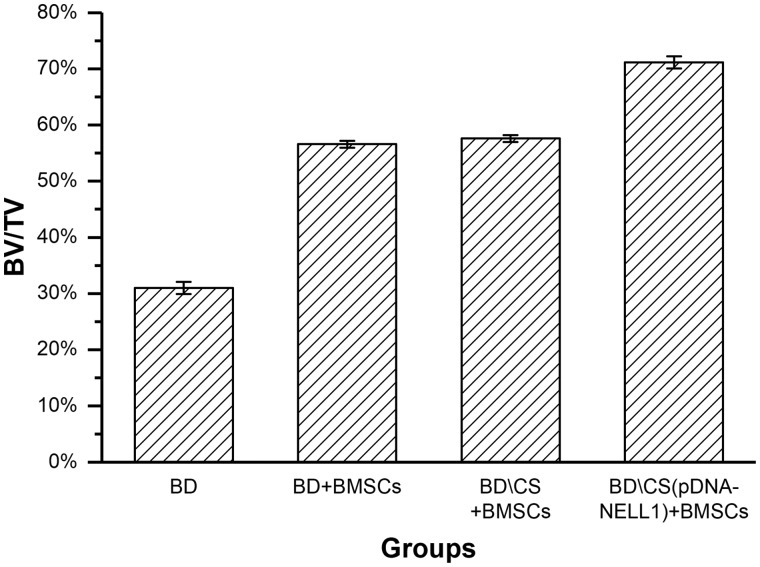
The bone volume of new alveolar sockets in each group at 12-week post-operation

By calculating, the ratio of bone volume and sample tissue volume (BV/TV) for each group was quantitatively determined. As shown by Fig. 8, *P* values between any two groups were < 0.05, except that between BD+BMSCs and BD\CS + BMSCs groups. Therefore, the regenerated bone of the BD\CS(pDNA-NELL1) + BMSCs group was the maximum among all the groups.

### Histopathological observations

**Figure 9:**
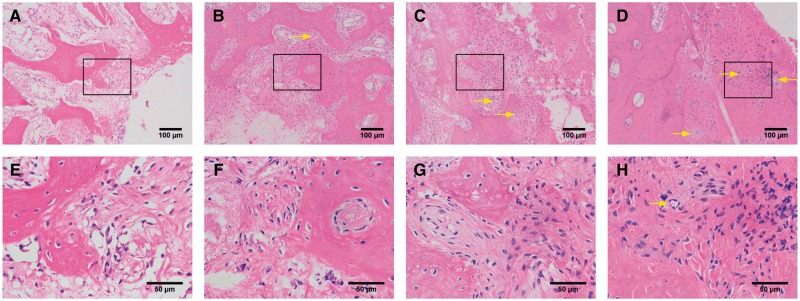
Histopathological observations of bone regeneration at 12-week post-operation by HE staining. **(A)** and **(E)** for the BD group, **(B)** and **(F)** for the BD+ BMSCs group, **(C)** and **(G)** for the BD\CS + BMSCs group, and **(D)** and **(H)** for the BD\CS(pDNA-NELL1) + BMSCs group, yellow arrows for new vessels. (A–D) and (E–H) are different magnifications, and (E–H) are the amplification of the boxes in (A–D), respectively

As shown by Fig. 9, hematoxylin–eosin (HE) stained tissue sections at 12-week post-operation revealed that the new bone was completely fused with the host bone in the experimental group (BD\CSn(pDNA-NELL1) + BMSCs), while the scaffold was completely resorbed. Dense new bone with regular osseous lamella has formed, and there was a large number of mature osteocytes and new vessels (Fig. 9D and H). New bone in the control Group 1 (BD) exhibited several cavities and connective tissue with unabsorbed scaffolds, and few osteocytes formed, which were sparsely distributed with inflammatory cell infiltration (Fig. 9A and E). The density and quantity of the new bone were lower in the BD + BMSCs (control Group 2, Fig. 9B and F) and BD\CSn + BMSCs (control Group 3, Fig. 9C and G) groups than those of the experimental group, but higher than those of the control Group 1 (BD), and had fewer number of cavities and connective tissue than that of the control Group 1 (BD). Unabsorbed scaffolds with macrophages on the edge were also observed in control Groups 2 and 3.

## Discussion

Alveolar bone defect repair presents an urgent problem for dentists. The theory of tissue engineering can be used to design novel treatment strategies for the repair of large bone defects. According to the theory of tissue engineering, a tissue engineered bone includes the following three elements: (i) a scaffold with good histocompatibility; (ii) a sufficient number of seed cells; and (iii) growth factors [3, 15–17].

BG exhibits good bioactivity, degradation, and the ability to promote the proliferation of osteocytes; thus, it has become a typical representative of the third generation of biomaterials used to repair bone defects [18]. In particular, sol-gel techniques provide a new method for the preparation of porous bioglass. In previous related studies, nanoscale BG demonstrated rapid and dense cell adhesion property, and promoted bone regeneration and angiogenesis [19, 20]. Recently, 3D printing technology (e.g. solid free forming manufacturing technology), also known as rapid prototyping technology or additive manufacturing technology, has been gaining absolute superiority in scaffold manufacturing due to its highly accurate plasticity [21]. This study adopts the preparation of BG scaffold by the most advanced 3D printing technology, with a multi-layer pore structure and function bearing surface; the BGs can be processed according to particular needs with high accuracy. Moreover, 3D printed BG exhibits significantly improved compressive strength compared with traditional sintered porous hydroxyapatite scaffolds, and the nano structure and the bioactive components loaded by the scaffold efficiently and cooperatively improve bone formation [22].

As an essential factor of tissue engineering, seed cells are required in this study. BMSCs are stem cells with a high reproduction rate and multi-directional differentiation potential in the bone marrow, which can differentiate into osteoblasts, chondrocytes, and other cell types under appropriate conditions. Thus, BMSCs are ideal seed cells in bone tissue engineering, and are currently one of the most widely studied seed cells [23–26]. In addition, BMSCs have the ability to differentiate into three blastoderms, all of which exhibit the effects of healing bone defects via *in vitro* induction or direct implantation of the bone defect region [27–29]; however, if the local environment lacks bone growth factor, the osteogenic effect is not ideal [27]. Although, NELL1 and its role in bone tissue engineering remains poorly understood, osteogenic activity of NELL1 has been confirmed by animal experiments, and biomaterials containing NELL1 can promote rat cranial defect repair. Therefore, with an appropriate scaffold as carrier and an effective controlled release system, NELL1 is a novel effective osteogenic gene and an useful factor in bone tissue engineering [8, 9]. CS is the only cationic natural polysaccharide currently available that has a similar chemical structure as the extracellular matrix, glycosaminoglycan and has favorable biocompatibility, biodegradable properties, and biological activity. CS can be combined with DNA nanoparticles to achieve controlled release of the DNA, and to perform targeted DNA delivery [30–32].

Based on above mentioned advantages of BG scaffold and CS, this study used 3D printing technology to prepare porous BG scaffold, which were activated by a CS composition that carried pDNA-NELL1 recombinant plasmid. Such composite provided favorable environment for the adhesion, migration, proliferation, and differentiation of BMSCs.

Since rhesus monkeys are closely related to humans as a species, anatomical physiology and the functional status of the periodontal system, they were selected as the experimental animals in this study [33]. The rhesus monkey model is highly representative of human beings, and the results obtained from this model have substantial credibility and are of guiding significance for clinical treatment. In this study, the regeneration of alveolar bone in the defect region was routinely inspected by X-ray and micro-CT observations and measurements following the operation. The results revealed different levels of alveolar bone regeneration in the four groups, while the BD\CSn(pDNA-NELL1) + BMSCs group obtained the best result among all the groups. As an important technique used to observe the basic structures of bone tissue, HE staining of the BD\CSn(pDNA-NELL1) + BMSCs group revealed the complete fusion of new bone and the host bone, a large number of mature osteocytes, and dense new bone; the structure of the new bone was similar to that of the normal bone tissue. Such results demonstrated that the BD\CSn(pDNA-NELL1) + BMSC group was associated with the best ability to repair alveolar bone defects in rhesus monkeys. The reason for this might be that the 3D printed BG scaffold had a micro/nano structure similar to that of the natural bone tissues, thus providing appropriate microenvironment for bone regeneration. The micro/nano structure of the BG scaffold was easily composited with CS nanoparticles, to promote NELL1 gene transfection and expression within the cell. This up-regulated the expressions of osteogenesis-related genes and proteins, promotes the secretion of extracellular matrix by BMCSs, and the formation of mineralized nodules.

## Conclusion

In conclusion, 3D printed BG scaffold loaded with NELL1 gene exhibited good biocompatibility, and is beneficial to the growth and adhesion of BMSCs, which were connected to each other through the porous structure. Therefore, such porous scaffold can provide good conditions in terms of nutrition intake, cell adhesion, and growth space for the formation of bone tissue. NELL1 gene further promoted the repair and regeneration of alveolar bone. Further study is required to completely regenerate the bone tissue defects in the periodontal tissue, as well as explore the mechanism of the NELL1 gene on promotion of the bone regeneration.

## Funding

This work is in part supported by China Postdoctoral Science Foundation funded project (No. 2013M540787).

*Conflict of interest statement*. None declared.
