# Correction to: Tissue engineering using 3D printed nano-bioactive glass loaded with NELL1 gene for repairing alveolar bone defects

**DOI:** 10.1093/rb/rbad065

**Published:** 2023-07-04

This is a correction to: Jing Zhang, Yang Chen, Jing Xu, Jingjing Wang, Chengzhang Li, Liyan Wang, Tissue engineering using 3D printed nano-bioactive glass loaded with NELL1 gene for repairing alveolar bone defects, *Regenerative Biomaterials*, Volume 5, Issue 4, August 2018, Pages 213–220, https://doi.org/10.1093/rb/rby015

In the originally published online version of this manuscript, the following sentence on page 215: “The animal experiments were approved by the Ethical Committee of Foshan Woman and Children’s Hospital.” should instead read: “The animal experiments were approved by the institutional Animal Care and Use Committee of Guangdong Landau Biotechnology Co. Ltd.”

This error has been outlined only in this correction notice to preserve the version of record.

